# Prey preference and life tables of the predatory mite *Parasitus bituberosus* (Acari: Parasitidae) when offered various prey combinations

**DOI:** 10.1007/s10493-013-9701-y

**Published:** 2013-05-03

**Authors:** Piotr Szafranek, Mariusz Lewandowski, Marcin Kozak

**Affiliations:** 1 Department of Applied Entomology, Faculty of Horticulture, Biotechnology and Landscape Architecture, Warsaw University of Life Sciences—SGGW, Nowoursynowska 159, 02-776 Warsaw, Poland; 2 Present Address: Research Institute of Horticulture, Konstytucji 3 Maja 1/3, 96-100 Skierniewice, Poland; 3 Department of Botany, Faculty of Agriculture and Biology, Warsaw University of Life Sciences—SGGW, Nowoursynowska 159, 02-776 Warsaw, Poland

**Keywords:** Mushroom pest, Biological control, Prey choice test, Population parameters

## Abstract

*Parasitus bituberosus* Karg (Acari: Parasitidae) is one of the predatory mite species inhabiting mushroom houses. It is known to accept a wide range of prey, suggesting that it may be a promising candidate for the biological control of key pests of mushroom culture. In our study it did not show any prey preference among four groups of small organisms often occurring in mushroom growth medium, namely rhabditid nematodes, pygmephorid mites, and sciarid and phorid fly larvae. Nevertheless, the type of food these predators fed on affects their development. The shortest egg-to-adult development time was obtained on a nematode diet. On a diet of phorid larvae, mite development stopped at the deutonymph stage; none reached adulthood. All other diets sufficed to reach the adult phase. Female fecundity when fed nematodes and sciarid larvae did not differ, but it was much lower when fed pygmephorid mites. Other life table parameters confirmed that pygmephorid mites constituted the worst diet for *P. bituberosus*. The highest intrinsic rate of population increase (*r*_*m*_ = 0.34) was obtained on the nematode diet; when fed sciarid larvae and pygmephorid mites it was 0.25 and 0.14, respectively. Our study provides good reasons to further test *P. bituberosus* as biocontrol agent of especially sciarid flies and nematodes, especially when the compost is well colonized by mushroom mycelium (which retards nematode growth).

## Introduction

The mushroom environment with its large amount of decaying organic material, mycelium and sporophores is favourable for many small animals. Some of them can feed on the mushrooms and can be treated as pests. Others, mainly predatory mites, can feed on these pests, limiting their populations. Studies conducted in mushroom houses in Poland in the 1980s showed the occurrence of 19 mite species belonging to eight families. Among them 11 predatory species, from four families: Macrochelidae, Eviphididae, Ascidae and Rhodacaridae (Kropczyńska-Linkiewicz 1984). Twenty years later, also the parasitid *Rhabdocarpais* *consanguineus* (Oudemans and Voigts) (syn. *Parasitus consanguineus* Oudemans and Voigts) was found in a few Polish mushroom houses (Szlendak and Lewandowski 2000). This species was first found in mushroom houses by Gill et al. (1988) and Trivedi (1988). Another species of the Parasitidae family, *Parasitus bituberosus* Karg, was noted in cultivated mushroom beds by Binns (1973a) and Al-Amidi and Downes (1990).

A few insect species were listed as the main pests of mushroom culture, causing direct and indirect damage by feeding on mycelium and mushroom sporophores, contaminating the crop and transmitting spores of mushroom diseases (Hussey et al. 1969; White 1981, 1986; Rinker and Snetsinger 1984). Among the mites occurring in mushroom houses, species from the Acaridae, Tarsonemidae, Siteroptidae and Histiostomatidae were mentioned as economically important (Hussey et al. 1969). The most common is *Pediculaster mesembrinae* (Canestrini), which can thrive while feeding on weed moulds, but it is unable to feed and reproduce on substrate colonized by mushroom mycelium. However, the mites can indirectly injure mushroom culture by favoring the development of the parasitic fungi *Trichoderma harzianum* and *T. viride*. Simultaneous infestation of *P. mesembrinae* and *Histiostoma feroniarum* (Dufour) resulted in 20–40 % reduction in mushroom yield (Clift and Terras 1995). In a group of nematodes infesting mushroom houses, the most destructive species can feed on mushroom mycelium, causing significant reductions in yield. Symptoms that can be noticed after their feeding are bare patches or sunken areas of bed. Fortunately, these species are not as common in the mushroom environment as bacterivorous rhabditid nematodes. In Polish mushroom houses, 90 % of all nematodes found in casing were represented by *Rhabditis cucumeris* Marcinowski. Presence of the mites in the compost delayed development of mushroom mycelium (Dmowska et al. 1997).

Chemical control of dipteran pests associated with the cultivation of mushrooms is unsatisfactory, which has led to an increasing interest in biological control (Al-Amidi and Downes 1990). Usage of predatory mites for mushroom pest control was initiated by Binns (1972, 1973b, 1974), who studied mites phoretic on mushroom flies. He found that *Digamasellus fallax* (Leitner), phoretic on sciarid flies, accepts nematodes as food. Equally common *Arctoseius cetratus* (Sellnick), also phoretic on flies, can attack sciarid larvae and eggs, cecid larvae, tarsonemid mites and rhabditid nematodes. Another *Arctoseius* species, *A. semiscissus* (Berlese), was recorded on reared mushrooms in Poland by Dmoch (1995), who considered this phoretic mite a potential agent for sciarid fly control. Soil-dwelling mites are another group studied for their potential use in biological control. *Hypoaspis miles* (Berlese) and *H. aculeifer* (Canestrini) were proven effective against sciarids of the genus *Bradysia* (Gillespie and Quiring 1990; Chambers et al. 1993; Wright and Chambers 1994), *Lycoriella ingenua* (Dufour) (Enkegaard et al. 1997; Jess and Bingham 2004) and the phorid *Megaselia halterata* (Wood) (Jess and Bingham 2004).

Mushroom substrate based on peat, manure and straw may constitute a perfect environment for *P. bituberosus* (Hyatt 1980). In mushroom beds it can find much of various kinds of food, such as nematodes, saprophagous and mycophagous mites, springtails, and dipteran larvae of the families Sciaridae, Phoridae and Cecidomyiidae (Hussey et al. 1969; Al-Amidi and Downes 1990; Al-Amidi et al. 1991). Used as a biological control agent of dipteran larvae, *P. bituberosus* prevented the cecidomyiid *Heteropeza pygmaea* Winertz from building up high populations. It was also able to limit populations of the sciarid *L. ingenua* by 50–66 %. Larvae of *L. ingenua* and *M. halterata* were also accepted as a prey by *R. consanguineus*. Studies showed that *R. consanguineus* can complete development on a diet of larvae of *M. halterata* and *L. ingenua*, which indicates that it has potential as a predatory mite for controlling both these pest species (Szlendak and Lewandowski 2009). The studies mentioned suggest that predatory mites in mushroom houses can be considered as natural enemies of mushroom pests, and that some of them may replace chemicals in mushroom pest control.

According to Al-Amidi and Downes (1990) and Al-Amidi et al. (1991), one of the most interesting species seems to be *P. bituberosus*. To date little is known about its biology and influence on mushroom yield. Al-Amidi et al. (1991) found that sciarid fly control by these mites led to an 18 % increase in mushroom yield. Considering the relatively large size of the mites, it will also be interesting to study its influence on transmitting spores of mushroom diseases and mushroom crop quality. Here, as a first step of ongoing investigations, we study the influence of prey types on the biology of *P. bituberosus*. Specifically, we study (a) prey preferences of *P. bituberosus* feeding on some small animals commonly occurring as pests in mushroom houses, viz. rhabditid nematodes, pygmephorid mites and dipteran larvae, and (b) the influence of the food on development of the mites. Such knowledge will constitute a basis for further research on the role of *P. bituberosus* in mushroom production.

## Materials and methods

Specimens of *P. bituberosus* for our experiments were obtained from a stock colony that had been established with mites collected in 2005 from two commercial mushroom houses located near Warsaw, Poland. Colonies were maintained in the laboratory at 25 °C in six plastic boxes filled to 1/3 height with horse dung. Rhabditid nematodes were added to the boxes as prey. For keeping colonies in good condition, every 2 weeks two new boxes were prepared and the two oldest were removed.

Four types of diets were used to study prey preferences of *P. bituberosus*: (1) bacterivorous nematodes of *Rhabditis* sp., (2) 2nd instar larvae of the sciarid fly *L. ingenua* (3) 2nd instar larvae of the phorid fly *M. halterata*, and (4) all developmental stages of the fungivorous mite *P. mesembrinae*. Prey preferences of starving females and deutonymphs were studied in choice tests conducted in a plastic arena. The prey were placed separately in small plastic cups (8 mm diameter and 5 mm high). To one cup, a small amount of compost inoculated with mushroom mycelium was added along with one larva of sciarid or phorid fly, 10 females of pygmephorid mites, or about 300 individuals of rhabditid nematodes. Higher numbers of pygmephorid mites and nematodes were added than fly larvae, to roughly equalize the amount of biomass of available prey. Plastic Petri dishes (10 cm diameter) were used as an arena. At the bottom of the Petri dish, wet blotting paper was laid to prevent the mites to desiccate. 4 cups, containing different diets, were placed symmetrically inside Petri dishes, and one individual of *P. bituberosus* starving for 24 h was placed in the centre of the dish. A new arena was constructed for each predator specimen examined. The kind of prey chosen, time of searching the prey and time of feeding were scored. The predatory mites were observed until they ended feeding; if a specimen did not choose any cup within an hour or if any prey left the cup (thereby confounding the set-up), it was rejected. When a predator specimen entered a cup and started feeding, this was treated as choosing the food and as the end of searching time; leaving the cup after eating one or more specimens of prey was treated as the end of feeding. If they visited a cup and left it without feeding, searching time was measured until the moment of feeding on some other prey. The tests were conducted for 30 females and 42 deutonymphs, used only once in the test.

### Life table parameters

The experiments were conducted in glass rearing cages (4.0 × 3.5 × 0.5 cm) with a centrally drilled conical hole of 7–15 mm diameter. Filter paper was attached on one side of the cage, using hot wax as an adhesive. The other side of the cage was covered with a microscope slide cover slip attached to the glass by strips of sticky label tape.

One pair of *P. bituberosus* was placed in each cage, with a small amount of horse dung and nematodes as a diet. After 24 h, eggs of *P. bituberosus* were selected under a stereo-microscope, and put separately in rearing cages filled with one of the four diets, used in prey preferences tests: (1) rhabditid nematodes, (2) 1st or 2nd instar sciarid larvae, (3) 1st or 2nd instar phorid larvae, or (4) all developmental stages of pygmephorid mites. The amount of prey supplied was in excess of what the predators eat maximally. The rearing cages were placed in controlled-temperature cabinets maintained at 25 (± 0.5) °C and 12 h daily photophase.

Fifty eggs of *P. bituberosus* were used for each food type. Fresh food was given to the rearing cages every day. After reaching the deutonymph stage, mites were moved separately to plastic boxes of about 130 ml capacity, filled with about 10 ml of horse dung and the corresponding diet, to moult into adults. The viability of eggs, larvae, proto- and deutonymphs as well as the duration of their development was established by daily recording the number of live and dead individuals. Mortality, longevity and fecundity of females were also recorded by placing one-day-old pairs of *P. bituberosus* in separate, small rearing cages, replaced daily by new ones, containing fresh food. Because multiple mating increases egg production (Yasui 1997), a male was replaced if it died earlier than the female. To obtain more data on female development parameters, an additional series of experiments was established with an additional group of *P. bituberosus*, reared under the same conditions and on the same type of diet as in the main experiment.

### Statistical analysis

Results of choice tests were analyzed through the χ^2^ test for analyzing frequencies (Agresti 2002). Time of searching the prey and time of feeding were analyzed through analysis of variance; in case of lack of fit of the analysis of variance model, as suggested by graphical diagnostics methods, generalized least square estimation was employed with a best-fit variance function (Pinheiro and Bates 2000), with the help of ‘nlme’ package of R. For significant models, Tukey’s contrasts without adjustment for multiple testing were employed. Survival rate of *P. bituberosus* on different diets for subsequent stages was analyzed through generalized linear models with a binomial error distribution (Agresti 2002). Tukey’s contrasts for generalized linear models (Hothorn et al. 2008) were employed to compare the diets in terms of survival rate on different diets (the diets for which *l*_*x*_ = 0 were removed from the multiple comparisons); no adjustment was made for multiple testing (Webster 2007; Kozak 2009). The significance level for all the analyses was 0.05. The analysis was conducted in R (R Development Core Team 2009); for multiple comparisons, the package ‘multcomp’ (Hothorn et al. 2008) was employed. Differences of the stages among diets in mean development time, longevity of females, number of eggs laid, and duration of oviposition period were studied in the same way as time of searching the prey and time of feeding, as described above.

Life tables were constructed from the observed age-specific survival rate (*l*_*x*_) and age-specific fecundity rate (*m*_*x*_) [*R*_*0*_, net reproductive rate (female progeny per female); *T*, mean generation time (days); *r*_*m*_, intrinsic rate of population increase (female per female per day); *λ*, finite rate of population increase (female per female per day)] (Birch 1948). For estimation of standard errors and multiple comparison of the diets in terms of these life table parameters, the jackknife method was employed (Maia et al. 2000), without adjustment for multiple testing.

Age-specific survival rate of *P. bituberosus* females on different food diets was analyzed as a Weibull function of day (Pinder et al. 1978), fitted by means of a nonlinear least squares method, using R.

## Results

### Prey preferences

Frequency analysis did not show any differences in choice of particular food diets of females (*χ* ^*2*^ = 0.667; *df* = 3; *P* = 0.84) and deutonymphs (*χ* ^*2*^ = 0.857; *df* = 3; *P* = 0.88) of the predatory mite *P. bituberosus*.

Time of searching the prey was determined only by developmental stage (*P* < 0.001); the effects of prey and interaction were not significant (*P* = 0.46 and 0.94, respectively). Females reached the prey much faster (with the mean of 6.0 min) than deutonymphs (17.3 min).

Time of feeding was determined only by prey (*P* = 0.004); the effect of neither developmental stage (*P* = 0.71) nor interaction (*P* = 0.42) was significant. The mean time of feeding was the shortest for nematodes, and the longest for phorid and sciarid larvae (Table 1).

**Table 1:** Mean (±SD) time of searching the prey and time of feeding for two stages of *Parasitus bituberosus* on different food sources

Prey	Searching time				Feeding time			
	n	Female	n	Deutonymph	n	Female	n	Deutonymph
Rhabditid nematodes	9	4.1 ± 1.3aA	12	18.2 ± 17.5aB	9	13.4 ± 1.3aA	12	9.2 ± 5.2aA
Pygmephorid mites	6	4.7 ± 4.5aA	9	12.2 ± 7.7aB	6	15.5 ± 7.6abA	9	15.3 ± 10.0abA
Sciarid larvae	8	6.6 ± 6.6aA	12	20.3 ± 14.6aB	8	19.0 ± 11.1bA	12	23.9 ± 14.5bA
Phorid larvae	7	8.7 ± 8.1aA	9	17.7 ± 7.3aB	7	15.0 ± 6.4bA	9	19.4 ± 4.4bA

Means within a column followed by different lower-case letters, and means within a row followed by different upper-case letters, are significantly different (*P* < 0.05)

### Life table parameters

Predatory mites *P. bituberosus* were able to complete their life cycle on all the food diets except phorid fly larvae, for which no adult individuals were obtained during the experiments. The age-specific survival rate of immatures differed among diets (*P* = 0.017), but no significant differences were observed at the beginning of the deutonymph stage class. The worst diet was phorid larvae, for which the parameter reached zero at the beginning of the adult age class (Table 2).

**Table 2:** Survival and mortality tables of *Parasitus bituberosus* on different food sources

Predator stage	Prey type	No. alive at start of stage interval (*n*_*x*_)	Proportion survived at start of stage interval (*l*_*x*_)	No. died within stage interval X to X + 1 (*d*_*x*_)	Finite rate of mortality (*q*_*x*_)
Eggs	Rhabditid nematodes	50	1.0a	0	0.0
	Pygmephorid mites	49	1.0a	16	0.33
	Sciarid larvae	50	1.0a	9	0.18
	Phorid larvae	49	1.0a	5	0.10
Larvae	Rhabditid nematodes	50	1.0a	7	0.14
	Pygmephorid mites	33	0.67c	7	0.21
	Sciarid larvae	41	0.82bc	9	0.22
	Phorid larvae	44	0.90b	7	0.16
Protonymphs	Rhabditid nematodes	43	0.86a	6	0.14
	Pygmephorid mites	26	0.53b	2	0.08
	Sciarid larvae	32	0.64ab	3	0.09
	Phorid larvae	37	0.76ab	9	0.24
Deutonymphs	Rhabditid nematodes	37	0.74a	6	0.16
	Pygmephorid mites	24	0.49a	6	0.25
	Sciarid larvae	29	0.58a	5	0.17
	Phorid larvae	28	0.57a	28	1.0
Adults	Rhabditid nematodes	31	0.62a	31	1.0
	Pygmephorid mites	18	0.37b	18	1.0
	Sciarid larvae	24	0.48ab	24	1.0
	Phorid larvae	0	0.0	0	0.0

Means within a predator stage followed by different letters are significantly different according to multiple comparisons for the corresponding model (see text) (*P* < 0.05)

Development time of preimaginal stages (from egg to adult) of *P. bituberosus* significantly differed among diets (*P* ≤ 0.001); nematodes constituted the best food, on which immature stages of the mites developed faster than on the other diets (Table 3).

**Table 3:** Mean (±SD) developmental time (days) of immature stages of *Parasitus bituberosus* on different food sources

Prey	Stages													
	n	Eggs^1^	n	Larvae	n	Protonymph	n	Deutonymph	n	Egg-to-adult	Egg-to-adult			
											n	Female	n	Male
Rhabditid nematodes	50	1.1 (45)	43	1.1 ± 0.2a	37	1.0 ± 0.2a	31	2.7 ± 0.7a	31	5.9 ± 0.8a	15	6.1 ± 1.0a	16	5.6 ± 0.7a
Pygmephorid mites	33	1.1 (30)	26	1.2 ± 0.4a	24	1.4 ± 0.5b	18	4.1 ± 1.8b	18	7.7 ± 1.7b	10	8.4 ± 1.8b	8	6.8 ± 1.2a
Sciarid larvae	41	1.0 (40)	32	1.5 ± 0.5b	29	1.9 ± 0.8bc	24	3.3 ± 1.9ab	24	7.7 ± 1.9b	7	6.6 ± 1.0a	17	8.1 ± 2.0b
Phorid larvae	44	1.0 (44)	37	1.1 ± 0.3a	28	1.6 ± 0.5c	28	4.4 ± 5.4^2^	No adults					
*P* value	–		0.001		0.001		0.005		0.001		0.004		<0.001	

Means within a column followed by different letters are significantly different according to multiple comparisons for the corresponding model (see text) (*P* < 0.05)

^1^For eggs the number of eggs for which developmental time was 1 day was placed in parenthesis; for the other eggs, the time was 2 days

^2^For deutonymphs, developmental time on a diet of phorid larvae was calculated to the moment the deutonymphs died. This group was not included in the statistical analysis, hence it is not accompanied by a letter

Diets did not affect *P. bituberosus* female longevity (*P* = 0.19), but affected total fecundity (*P* < 0.001). The lowest fecundity was observed for females fed upon pygmephorid mites, whereas no difference was detected between fecundity of females fed upon rhabditid nematodes and sciarid larvae. The same situation was observed for daily fecundity (*P* < 0.001). Because of an incomplete life cycle of *P. bituberosus* on phorid larvae, this diet was not included in the statistical analyses. The duration of oviposition period, like longevity, was independent of the type of food (Table 4). Because most females—about 95 %—laid eggs on the day of pairing and copulation with a male, and 85 % of them died on the day of laying the last eggs or the next day, the analyses did not include the data on duration of pre- and postoviposition period.

**Table 4:** Mean (±SD) female longevity (days), duration of the oviposition period (days), total fecundity (eggs/female), daily fecundity (eggs/female/oviposition day) and sex ratio (female/female + male) of *Parasitus bituberosus* on different food sources

Prey	n	Longevity of females	Oviposition period	Total fecundity	Daily fecundity	Sex ratio
Rhabditid nematodes	30	6.5 ± 2.5a	5.4 ± 2.6a	59.5 ± 33.1a	10.4 ± 4.2a	0.48
Pygmephorid mites	28	5.4 ± 1.5a	4.2 ± 1.9a	18.1 ± 11.6b	4.2 ± 2.1b	0.58
Sciarid larvae	21	5.8 ± 3.0a	5.4 ± 2.9a	51.3 ± 34.2a	8.9 ± 3.4a	0.50
Phorid larvae	No adults					

No adults developed on *Megaselia halterata* diet, so this group was not considered in statistical analyses

Means within a column followed by different letters are significantly different according to multiple comparisons for the corresponding model (see text) (*P* < 0.05)

Life-history data obtained from daily observations were used to construct life tables (Table 5). The intrinsic rate of natural increase (*r*_*m*_), fine rate of increase (*λ*) and net reproductive rate (*R*_*0*_) were highest for the nematode diet. Sciarid larvae turned out to be a worse diet than nematodes and better than pygmephorid mites. Differences in the mean values of those three parameters were significant for all diets, except for phorid larvae, a diet on which no adult predators were obtained. Mean generation time (*T*) was the shortest for *P. bituberosus* fed on nematodes, no differences were detected among the other diets.

**Table 5:** Mean (±SE) life table parameters of *Parasitus bituberosus* on different food sources and multiple comparisons obtained by the jackknife method

Prey	n	*R*_*0*_	*T*	*r*_*m*_	*λ*
Rhabditid nematodes	30	17.7 ± 1.80c	8.5 ± 0.27a	0.34 ± 0.01c	1.40 ± 0.02c
Pygmephorid mites	28	3.9 ± 0.47a	9.9 ± 0.21b	0.14 ± 0.01a	1.15 ± 0.01a
Sciarid larvae	21	12.3 ± 1.79b	9.8 ± 0.35b	0.25 ± 0.01b	1.29 ± 0.01b
Phorid larvae	no adults				

Means within a column followed by different letters are significantly different, according to multiple comparisons, computed by the jackknife method (*P* < 0.05)

*R*_*0*_ net reproductive rate, *T* mean generation time, *r*_*m*_ intrinsic rate of population increase, *λ* finite rate of population increase per female per day

**Fig. 1:**
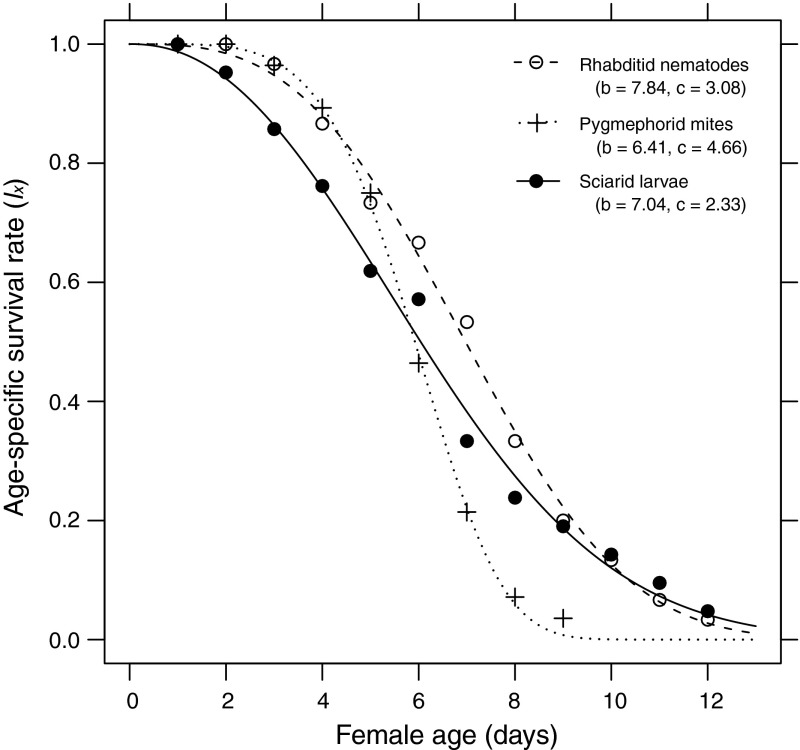
Age-specific survival (*l*_*x*_) of *Parasitus bituberosus* females on three diets. *Lines* represent fitted Weibull functions with *b* as scale and *c* as shape parameters

Distribution of the age-specific survival rate (*l*_*x*_) of female is presented in Fig. 1. These findings confirmed that in all of the prey species examined, the survival curve is type I, as parameter *c* describing the shape of the Weibull curve is higher than 1 (Pinder et al. 1978), but they were different in the scale parameter (*b*). For females reared on rhabditid nematodes and sciarid larvae, the survival curves had very similar shapes. The survival curve of females reared on pygmephorid larvae fell more sharply than the two other curves after day 4 of female life.

**Fig. 2:**
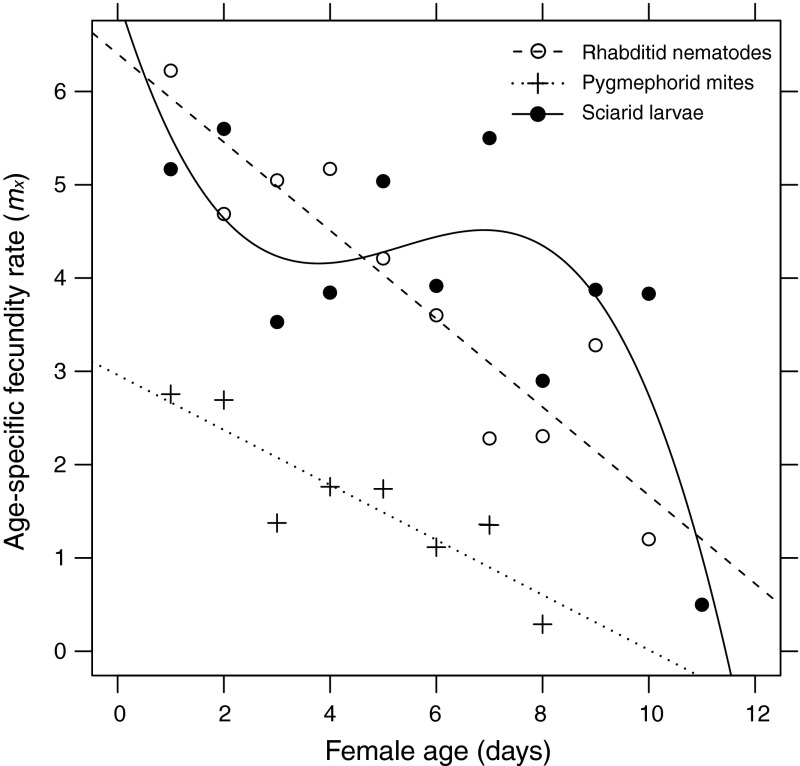
Age-specific fecundity of *Parasitus bituberosus* females on different food diets. *Lines* represent fitted relationship of *m*_*x*_ with female age

The age-specific fecundity curves (*m*_*x*_) for the diets differed in shape. For nematodes and pygmephorid mites the data fitted a linear function, representing a constant decrease of fecundity rate. Data for sciarid larvae diet fitted a third-order polynominal function; the curve was almost flat (i.e., parameter values were more or less constant) from the second to the eighth day in terms of female age since maturity (Fig. 2).

## Discussion

Prey acceptance tests of *P. bituberosus* have been conducted by Al-Amidi and Downes (1990). As a prey the authors used organisms commonly available in mushroom composts, such as larvae of cecid fly (*H. pygmea*), larvae and eggs of sciarid fly (*L. ingenua*), saprophagous mites (*H. feroniarum*), mycophagous mites (*Pygmephorus* sp. [=*Pediculaster*]) and springtails. Deutonymphs of the predator most frequently chose cecid and sciarid larvae as food, and seldomly sciarid eggs and springtails. In our studies the testing method and types of food were slightly different: the most common and economically important organisms in Polish mushroom houses were used, so that it was possible to evaluate which of them might be the main diet of this predator. Neither deutonymphs nor females showed preferences towards prey, which indicates that *P. bituberosus* in mushroom houses can feed on all invertebrates that inhabit growth medium used for mushroom cultivation. Very interesting are differences in choosing pygmephorid mites in our results and those obtained by Al-Amidi and Downes (1990). Al-Amidi and Downes did not observe deutonymphs feeding on *H. feroniarum* and *Pediculaster* sp., whereas our data demonstrated that starving deutonymphs chose pygmephorid mites as frequently as the other kinds of food. These differences can be a result of using normal and phoretic females of *P. mesembrinae* in the present study. We suspect that phoretic females, which are highly sclerotized, could be less accepted than normal ones. Unfortunately, Al-Amidi and Downes (1990) did not mention the stage of pygmephorid mites offered as a prey. An alternative explanation of these differences can be genetic differences in prey preferences among the studied populations of *P. bituberosus*. Such a phenomenon was observed in local populations of *Hypoaspis aculifer* (Canestrini) (Lesna and Sabelis 1999, 2002).

To find the prey deutonymphs needed twice as much time as females. According to our observations, deutonymphs in the phoretic stage are more active than females, and any perturbation of rearing boxes elicits activity of deutonymphs, which probably promotes searching for phoretic hosts and probably results from a motivational drive to reproduce (Al-Amidi and Downes 1990). Deutonymphs of this mite species experience periodic starvation, thereby prolonging the time of searching prey. Worth noticing is the significant difference in time of feeding on nematodes and dipteran larvae, as observed for both developmental stages of the predator. This was probably due to the difference in size of the prey: much smaller and more numerous than dipterans, nematodes were caught and eaten faster. The opposite situation was observed for sciarid and phorid larvae, which are much bigger than *P. bituberosus*—for this reason the predator needed much more time to attack and kill the prey.

Choice tests were also conducted by Rudzińska-Sajdak (1998) for *A. semiscissus*, which occurs in mushroom houses. The mites preferred nematodes and eggs and larvae of the sciarid *Lycoriella castanescens* (Lengersdorf), but did not show much interest in larvae of the cecidomyiid *H. pygmaea* or in acaroid *Tyrophagus putrescentiae* (Schrank) mites. Interestingly, Rudzińska-Sajdak’s (1998) choice tests showed a positive influence of the previous diet on prey choice. This phenomenon was not studied in the present study, but the fact that individuals used in the choice tests came from the cultivation on nematodes and yet did not choose this food most frequently may suggest that the association found by Rudzińska-Sajdak did not apply to our study. This, together with lack of differences in the choice of food, suggests that *P. bituberosus* is polyphagous, which agrees with Binns’ (1973a) observations, who found that in mushroom compost it accepts a wide range of prey, including eelworms, insect larvae and mites. The author claimed his observations dealt with *Parasitus fimetorum* (Berlese), but this identification was likely incorrect. Al-Amidi and Downes (1990) suggest that Binns’ identifications were probably based on Costa (1961). According to Hyatt (1988), Karg (1972) described *P. bituberosus* from larvae, protonymphs, deutonymphs, males and females collected from agricultural soils at Cottbus (East Germany) and Cape Town (South Africa). Karg also stated that *P. fimetorum* sensu Costa (1961) from Israel was not Berlese’s *fimetorum*, but was conspecific with *bituberosus*.

Despite the lack of preferences of *P. bituberosus* towards the food sources studied, it was noted that the mean developmental time of immature stages and fecundity of females were determined by differences in diet. Taking into account mean developmental time of immature stages—from egg to adult—nematodes turned out to be the best prey for these predator stages, whereas phorid larvae should be considered as the worst food. Immatures originating from eggs laid by females from a stock colony, feeding upon larvae of these flies, reached the deutonymph stage; however, after being moved to a new environment with the very same food, they did not reach maturity. Such late inhibition of immature development of *P. bituberosus* suggests that the negative influence of phorid larvae diet is not connected with quality of the food, but rather with the behaviour of either prey or predator. This phenomenon needs scrutiny in future experiments. One hypothesis is that the physiological step to maturity poses special nutritional requirements that may not be fulfilled by phorid larvae. Alternatively, Yasui (1997) hypothesized that immature predators need the presence of the other sex before they develop to maturity. However, this hypothesis was falsified by our observations that maturity was reached in absence of the other sex, yet there may still be an interaction with nutritional requirements (the first hypothesis).

The life tables show an effect of diets. With respect to intrinsic rate of population increase (*r*_*m*_) and mean generation time (*T*), nematodes were the best food for *P. bituberosus*. The second best food was sciarid larvae, whereas the worst diet involved phorid larvae, especially because the predators did not complete their development. Taking into account the size of the various types of food tested, one can suppose that nematodes, being the smallest, were the easiest to capture for the *P. bituberosus* immature stages, as can be inferred from the length of egg-to-adult developmental time (Table 3). Because of the big size of sciarid larvae as compared to nematodes, immature individuals needed more time and energy to kill the prey (Sabelis 1985, 1992). Such a conclusion may seem in conflict with the results on length of developmental time for immature stages of *P. bituberosus* fed on pygmephorid mites, because the prey is smaller than sciarid larvae. In this case, the time was similar to that for *P. bituberosus* fed on sciarids. Nonetheless, it should be taken into account that pygmephorid females and the youngest instars of sciarids have similar body size. Body length of females of *P. mesembrinae* ranges from 0.17 to 0.25 mm (Hussey et al. 1969; Camerik et al. 2006), whereas body length of the first larval instar of sciarids ranges from 0.36 to 1.40 mm (Lewandowski et al. 2004). In addition, life table parameters (Table 5) and fecundity (Table 4) suggest that, phorid larvae put aside, *P. mesembrinae* was the worst type of food also for females of *P. bituberosus*, even though the size of prey was not so important for adults of the predator. We observed that females were able to kill the third instar of phorid larvae or even the fourth instar of sciarid larvae, whose body length can reach even 3.8 and 7.9 mm, respectively (Lewandowski et al. 2004, 2012). The *r*_*m*_ values for females fed on this prey were more than twice smaller than for those fed on nematodes and sciarid larvae, and fecundity was almost three times smaller. Therefore, one could suppose that pygmephorid mites can be eaten by all developmental stages of the predator, but the nutritional value of this food is likely smaller than that of the two other types of food. Reasons for this phenomenon are difficult to establish, but supposedly it results from composition of nutrients or defensive (unpalatable) chemical compounds of the prey (Sabelis 1985, 1992).

Life table parameters of *P. bituberosus*, and especially intrinsic rate of population increase (*r*_*m*_), enable one to compare its bionomy with that of other species occurring in mushroom houses. Slightly smaller values of *r*_*m*_ (0.32 females/female/day) together with longer mean generation time (*T*) was observed for species *Proctolaelaps deleoni* Nawar, Childers and Abou-Setta, belonging to the family Ascidae, reared on nematodes at the same temperature (Nawar 1992). Smaller values of *r*_*m*_ (0.23) were also recorded by Rudzińska (1998) for *A. semiscissus*, which belongs to the same family as *P. deleoni*, fed upon eggs of sciarids. In this case, the parameter value could depend on the type of food and temperature, which was lower (20 °C) than in the present study. Sciarid larvae were used as food by Enkegaard et al. (1997) to study bionomy of *H. miles* in 20 °C. Intrinsic rate of population increase observed by these authors was surprisingly low (0.07). The high value of this parameter obtained during the present studies suggest that *P. bituberosus* is better adapted to the environment of mushroom houses than *A. semiscissus* and *H. miles*, which in turn might suggest *P. bituberosus* as a potential agent for mushroom pest control.

A comparison of life table parameters between *P. bituberosus* and other species of the Parasitidae family would be very interesting, but unfortunately such information is very scarce. The only available data are for *R. consanguineus* (Szlendak and Lewandowski 2009). This publication does not contain *r*_*m*_ values; the authors report fecundity, longevity and duration of oviposition period for the species reared on phorid and sciarid larvae. What was interesting in their experiment was that *R. consanguineus* completed its development on both these diets. Female longevity of *R. consanguineus* reared on sciarid larvae (mean of 6.9) was slightly longer than that for *P. bituberosus* (mean of 5.8) reared on the same diet, whereas duration of oviposition period was similar for both species (mean of 5.0 and 5.4 days, respectively). Worth noticing is that *R. consanguineus* was reared at a lower temperature (21 °C), which can be a reason for this difference. However, fecundity of *R. consanguineus* (17.8 eggs/female) was noticeably smaller as compared to that of *P. bituberosus* (51.3). Fecundity of *R. consanguineus* was similar to that obtained for *P. bituberosus* reared on pygmephorid mites, which were the worst food among those which allowed for complete development of the mites. The almost 3 times higher fecundity of those two prey species seems not to be affected by the differences in temperature during the experiments. Hence, inter-species differences among these predators are likely to be the case.

Unpublished data (Szafranek and Lewandowski) showed that *P. bituberosus* is common in mushroom houses in Poland: it was recorded in half of them. From the data obtained by Al-Amidi and Downes (1990) and Al-Amidi et al. (1991) it follows that the mites can feed on all small invertebrates that infest mushroom houses, thereby limiting their population density. Their potential for reducing sciarid larvae and rhabditid nematodes was proven by the high values of life table parameters. Intrinsic rate of natural increase (*r*_*m*_) of *P. bituberosus* fed on these prey species was higher than that for the other predatory mites studied under similar conditions (Nawar 1992; Enkegaard et al. 1997; Rudzińska 1998). A comparison of the parameters, especially *r*_*m*_, between the prey species and the predator would be very useful to confirm the idea of using *P. bituberosus* as an agent for biological control of mushroom pest. Roy et al. (2003) stated that *r*_*m*_ values can be useful in biological control practice as a means of selecting promising biocontrol candidates on the basis of their reproductive potential. According to Janssen and Sabelis (1992), Sabelis (1992) and Sabelis et al. (2002), theoretically a predator that has a population growth rate equal to or greater than its prey should effectively regulate its population (given a sufficiently high predator–prey ratio at introduction). Unfortunately, to the best of our knowledge life table parameters for *L. ingenua* are not available in the literature, although it is the main mushroom pest. Such information is available for the pygmephorid mite *P. mesembrinae*. Hernández-Abarca et al. (2005) presented values of *r*_*m*_ obtained for the mites reared in 25 °C on *T. harzianum*, which were slightly higher (0.17) than those calculated for *P. bituberosus* (0.14) reared on the *P. mesembrinae* served as a prey (see Table 5). This suggests that the predator may not be an effective agent for biological control of pygmephorid mites. Worth noticing is that the authors mentioned *T. hartzianum* as a non-suitable food source for the pest. Probably, *P. mesembrinae* feeding on other fungi, such as *Chrysonilia sitophila*, *Cladobotryum dendroides* or *Mycogone perniciosa*, can achieve higher values of these parameters, than *P. bituberosus*. Life table parameters for rhabditid nematodes were presented by Venette and Ferris (1997). These authors studied the influence of temperature on the finite rate of increase of a few species of bacterial-feeding nematodes, including *Rhabditis cucumeris*: at 25 °C it was ca. 1.00 which was lower than the 1.40 we found for *P. bituberosus*. At 20 °C, however, it increased more than twice (ca. 2.3), suggesting that in this lower temperature the efficiency of predatory mites can be noticeably lower.

It is still premature to rate *P. bituberosus* as an effective natural enemy of rhabditid nematodes and pygmephorid mites, because life table parameters do not seem to be totally convincing and because mushroom mycelium has a strong negative effect on populations of the pest (Gurney and Hussey 1967; Dmowska et al. 1997). Mushroom mycelium colonizing compost used for mushroom production absorbs much of free water and creates a bacteriostatic zone, which can inhibit development of these small animals (Hesling 1966). However, data presented by Al-Amidi and Downes (1990) and Al-Amidi et al. (1991) show *P. bituberosus* is effective against sciarid and cecidomyiid flies, and its introduction to mushroom beds increases mushroom yield and limits contamination of mushrooms by cecidomyiid larvae. Based on these results, Al-Amidi et al. (1991) recommended the species for use in integrated pest management. The results of the present research seem to confirm the validity of this hypothesis, especially in the case of sciarid fly control. Furthermore, the ability of *P. bituberosus* to prey on pygmephorid mites and rhabditid nematodes indicates that the predator can inhibit growth of this pest population when the growth medium is well colonized by mushroom mycelium.
